# Point‐of‐care viral load monitoring: outcomes from a decentralized HIV programme in Malawi

**DOI:** 10.1002/jia2.25387

**Published:** 2019-08-23

**Authors:** Sarala Nicholas, Elisabeth Poulet, Liselotte Wolters, Johanna Wapling, Ankur Rakesh, Isabel Amoros, Elisabeth Szumilin, Monique Gueguen, Birgit Schramm

**Affiliations:** ^1^ Epicentre Paris France; ^2^ Médecins Sans Frontières Chiradzulu Malawi; ^3^ Médecins Sans Frontières Paris France

**Keywords:** HIV Care Continuum, 90‐90‐90, Decentralization, HIV, Treatment monitoring, Health System Strengthening, Treatment failure

## Abstract

**Introduction:**

Routinely monitoring the HIV viral load (VL) of people living with HIV (PLHIV) on anti‐retroviral therapy (ART) facilitates intensive adherence counselling and faster ART regimen switch when treatment failure is indicated. Yet standard VL‐testing in centralized laboratories can be time‐intensive and logistically difficult in low‐resource settings. This paper evaluates the outcomes of the first four years of routine VL‐monitoring using Point‐of‐Care technology, implemented by Médecins Sans Frontières (MSF) in rural clinics in Malawi.

**Methods:**

We conducted a retrospective cohort analysis of patients eligible for routine VL‐ testing between 2013 and 2017 in four decentralized ART‐clinics and the district hospital in Chiradzulu, Malawi. We assessed VL‐testing coverage and the treatment failure cascade (from suspected failure (first VL>1000 copies/mL) to VL suppression post regimen switch). We used descriptive statistics and multivariate logistic regression to assess factors associated with suspected failure.

**Results and Discussion:**

Among 21,400 eligible patients, VL‐testing coverage was 85% and VL suppression was found in 89% of those tested. In the decentralized clinics, 88% of test results were reviewed on the same day as blood collection, whereas in the district hospital the median turnaround‐time for results was 85 days. Among first‐line ART patients with suspected failure (N = 1544), 30% suppressed (VL<1000 copies/mL), 35% were treatment failures (confirmed by subsequent VL‐testing) and 35% had incomplete VL follow‐up. Among treatment failures, 80% (N = 540) were switched to a second‐line regimen, with a higher switching rate in the decentralized clinics than in the district hospital (86% vs. 67%, *p* < 0.01) and a shorter median time‐to‐switch (6.8 months vs. 9.7 months, *p* < 0.01). Similarly, the post‐switch VL‐testing rate was markedly higher in the decentralized clinics (61% vs. 26%, *p* < 0.01). Overall, 79% of patients with a post‐switch VL‐test were suppressed.

**Conclusions:**

Viral load testing at the point‐of‐care in Chiradzulu, Malawi achieved high coverage and good drug regimen switch rates among those identified as treatment failures. In decentralized clinics, same‐day test results and shorter time‐to‐switch illustrated the game‐changing potential of POC‐based VL‐testing. Nevertheless, gaps were identified along all steps of the failure cascade. Regular staff training, continuous monitoring and creating demand are essential to the success of routine VL‐testing.

## 1 Introduction

Viral load (VL) testing is the gold standard approach for monitoring treatment effectiveness in HIV‐positive patients on anti‐retroviral therapy (ART) 1. VL suppression can be a performance indicator for ART programmes 2. Regular VL‐monitoring allows identification of suboptimal adherence, treatment failure and identification of patients whose ART regimen should be switched 3, 4, 5, 6, 7, 8. Knowing their VL status can motivate patients to adhere to treatment 9, 10, and can allocate virally suppressed patients into differentiated models of care 11, 12. Yet despite being associated with better treatment outcomes and the prevention of acquired drug resistance 6, 13, routine VL‐monitoring remains under‐utilized in resource‐limited settings 14, 15, 16, 17, 18, 19, 20, 21. It was estimated that less than 50% of people on ART in resource‐limited settings received an annual VL test in 2017 22.The test's cost and complexity remain barriers to its use, as does a lack of understanding by some clinicians about VL's long‐term patient benefits and its role in prolonging the longevity of treatment regimens 18, 21.

Centralized testing with dried blood spots (DBS), is a practical and efficient VL‐testing method, with the drawback of often lengthy result turnaround‐time (TAT) 23, 24, 25, 26, 27. Point‐of‐Care (POC)‐technology offers an attractive alternative that provides same‐day results for clinical decisions by bringing testing directly to the facility 16, 18, 19, 20, 24, 28, 29, 30, 31, 32, 33. Today, several POC‐VL technologies are in the pipeline or are becoming more accessible for low‐resource settings 34.

To create access to VL‐testing in decentralized settings in sub‐Saharan Africa, Médecins Sans Frontières (MSF) has been implementing routine VL‐monitoring in six countries mainly using dried blood spot (DBS) testing in centralized laboratories 35, 36. A Point‐of‐care VL testing approach was chosen in two projects: Arua District referral hospital (Uganda) and the HIV programme of Chiradzulu District (Malawi), using the first available VL POC‐ platform SAMBA I (“Simple Amplification Based Assay”). SAMBA I is a semi‐automated and robust assay (no need for air‐conditioning/functions up to 35°C, closed cartridge system, no susceptibility to dust, no cold‐chain required for tests kits, no toxic waste containing guanidine thiocyanate) that uses plasma specimens and provides semi‐quantitative test results (above or below 1000 HIV‐1 RNA copies/mL) within 125 minutes. The platform showed high accuracy compared to standard laboratory‐based methods 37, 38, 39, received CE‐marking in April 2016, and requires minimal technical‐operator training to perform few sample manipulation steps: plasma preparation, installation of sample tubes and cartridge elements in SAMBA‐Prep instrument for automated RNA‐extraction, transfer of extracted material to the SAMBA‐amp instrument and visual result read‐out 37, 38. The SAMBA I VL system was registered by the Pharmacy, Medicines and Poisons Board of Malawi in December 2011 following a multi‐site field trial including Malawi 38. The Ministry of Health approved the programmatic implementation of SAMBA I VL by MSF for routine VL‐monitoring in Chiradzulu District within the framework of the UNITAID‐funded HIV VL initiative. Before implementing the POC‐VL approach, treatment monitoring relied on CD4 count testing.

The objective of this analysis was to describe the outcomes of VL monitoring during the first four years of routine POC‐VL‐testing. It is, to our knowledge, the first published report of outcomes of routine viral load monitoring from real‐world clinical sites in sub‐Saharan Africa using a POC‐based approach.

## 2 Methods

### 2.1 POC‐VL implementation

MSF introduced the SAMBA I technology (Diagnostics for the Real World Ltd, Cambridge, UK) at point‐of‐care in decentralized clinics and in the district hospital (DHOS) of the rural, resource‐limited Chiradzulu District, in southern Malawi (17% HIV prevalence) 40.The definition of POC‐testing followed that of WHO and Schito *et al*. 22, 41, which refers to a diagnostic test (whichever technology) that is performed near the patient with a fast turnaround time that permits immediate use of results for patient management. Implementation was in a step‐wise manner starting in the DHOS laboratory (August 2013) and in simple “mini‐laboratories” in four decentralized clinics: Namitambo (August 2013), Bilal (May 2014), Mbulumbuzi and Namadzi (November 2014). In all five sites the VL‐platform was placed in direct proximity to the outpatient clinics to facilitate same‐day results. MSF hired and trained phlebotomists on blood collection and laboratory technicians on SAMBA I operation, and all clinical staff (MOH and MSF) were trained on applying the VL‐monitoring algorithm. At DHOS, MOH integrated VL‐monitoring into their existing system, which returned lab results to the patients at next scheduled visit. In the decentralized clinics MSF had more direct influence by adding MSF clinical staff to support and mentor MOH, and by ensuring staff presence in the afternoon to support same‐day result review. SAMBA I performance was monitored by participation in the CDC proficiency testing programme 42, 43, and by external quality control (EQC) on randomly selected frozen patient samples sent monthly to an external laboratory (First MSF Belgium HIV programme in Thyolo District using Biomerieux Nuclisens, then Dream Laboratory Blantyre using Abbott real‐time m2000). From September 2013 to December 2016: 70 results submitted to CDC showed 0.99 correlation with two gold‐standard RT‐qPCR assays; EQC samples (N = 2631) showed 95.4% concordance. The SAMBA I VL invalid rate was 0.4%.

### 2.2 Study design and population

This was a retrospective, descriptive analysis of an ART patient cohort accessing HIV VL‐testing for the first‐time using SAMBA I VL. The analysis included all VL‐eligible patients (on ART for > 3 months, and scheduled visit between the date of on‐site POC installation and analysis censorship on 30 June 2017). “Date of VL‐eligibility” was the date of the first scheduled visit in the study period. Patients with prior VL test results or on third‐line regimens were excluded.

### 2.3 VL‐testing protocol and treatment failure algorithm

Plasma was prepared from venous blood and tested with SAMBA I VL according to the manufacturer's instructions. The 2011 MoH guidelines 44 recommend VL‐testing at six and twenty‐four months after ART initiation or regimen switch, every two years thereafter (ART milestones), and targeted VL for clinical or immunological treatment failure 1. In addition, “catch‐up” VL‐testing was also done for patients who never had a prior VL but did not meet an ART milestone.

WHO defines treatment failure as two sequential VL ≥1000 copies/mL with enhanced adherence support provided between tests 1. The 2011 Malawi MoH guidelines differed from this definition: patients with VL ≥5000 copies/mL at their second VL test were considered treatment failures, while patients with a result of 1000 to 5000 copies/mL continued to receive enhanced adherence counselling (EAC), requiring a third test to confirm treatment failure. To reconcile these guideline differences, suspected failure patients (SAMBA I VL ≥1000 copies/mL) at MSF‐supported sites received two follow‐up VL tests (3‐months apart) and EAC. First‐line patients with three SAMBA I VL ≥1000 copies/mL results were considered treatment failures and eligible to switch ART regimens; second‐line patients classified as such required in addition a drug resistance test.

### 2.4 Outcomes and definitions

We defined “VL‐coverage” as the proportion of VL‐eligible patients receiving a first VL test during the study period. Result turnaround‐time (TAT) was the number of days between blood collection and test result review by clinicians with a patient. For the failure cascade, we reported the number and proportion of suspected failure patients: (1) receiving follow‐up VL tests, (2) failing treatment, (3) switching regimens and (4) receiving post‐switch testing within 18 months of a first VL‐test. Failure cascade sensitivity analysis included patients whose first VL‐test was <18 months from the date of analysis censorship. Retention in care was assessed 18 months after suspected failure and is defined as those alive, not transferred, or not missing a scheduled appointment by >2 months as per MOH definition of lost‐to‐follow‐up 45, 46.

### 2.5 Data collection and analysis

Patient‐level data were captured on paper forms at each visit (including socio‐demographic, ART regimen, clinical and immunological information) and entered into an individual patient follow‐up database (FUCHIA v.1.7.1, Epicentre, Paris, France) 47. The laboratory request form captured all steps from test order to result review, and data were entered into a dedicated POC‐VL electronic database (REDCap, Research Electronic Data Capture, Vanderbilt, USA) 48. VL test data and patient‐level data were linked using unique patient identifiers. We excluded 6% (2330/35,914) of VL tests: 1925 tests with unmatched or missing patient identifiers, 405 with duplicate, discrepant, invalid or missing test results.

Descriptive analyses used medians with interquartile ranges (IQR) or counts with proportions. Pearson chi‐squared or Kruskall Wallis tests assessed differences in patient characteristics at date VL‐eligible (POC‐site, ART regimen, sex, age, years on ART, WHO stage, prior CD4 cell count) and outcomes. Data unavailable in the patient database were reported as missing. Analyses were conducted using STATA v.13 49.

### 2.6 Ethics

Routine VL‐monitoring using SAMBA I POC‐VL obtained the approval and support of the Malawian Ministry of Health. Implementation and retrospective analyses were approved by the MSF Ethics Review Board as part of an overall multi‐country UNITAID‐funded proposal. A waiver from individual informed consent was granted for the collection and analysis of routine monitoring data. Unique alphanumeric codes were used to identify individual patients, and no patient identifiers were included in the analysis database.

## 3 Results

**Figure 1:**
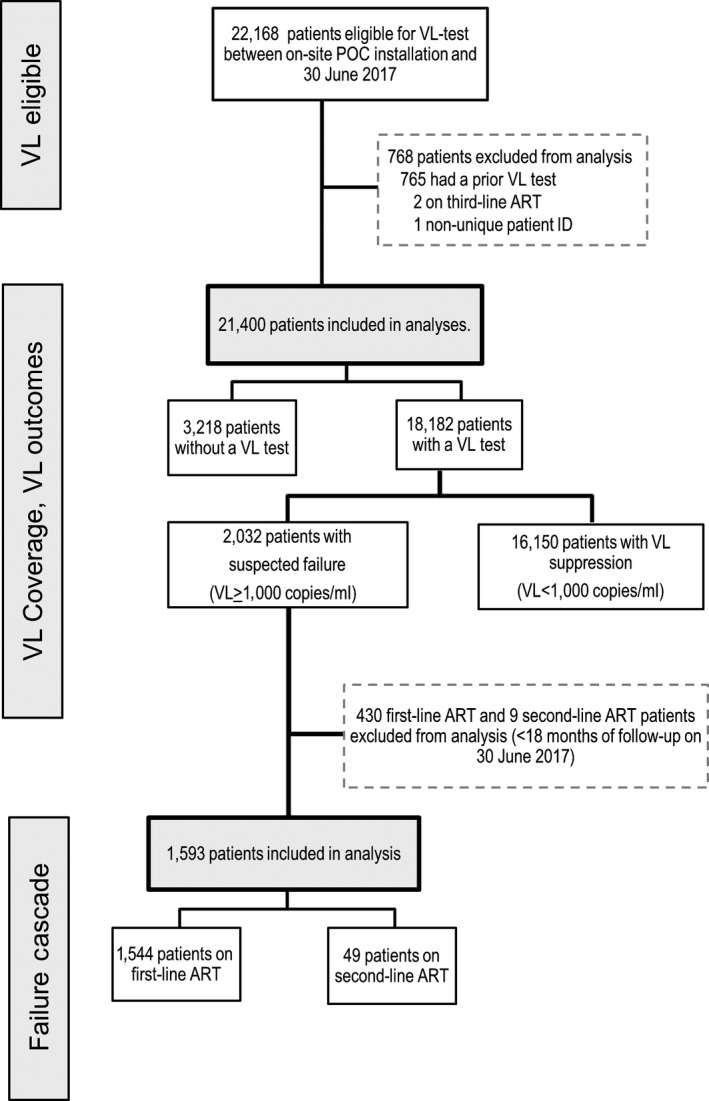
Flowchart of patients eligible for VL test and selected for analysis.

In total, 22,168 VL‐eligible patients were identified, of whom 21,400 (97%) were included in the analysis (Figure 1, Table 1). Of these, 91% were ≥20 years of age, 65% female, 48% had a cumulative WHO stage III or IV and 55% were on ART ≥2 years, with 98% on first‐line ART. Among those with available CD4 counts prior to VL‐eligibility, 66% had > 350 cells/μL.

**Table 1:** Characteristics of patients eligible for a VL‐test and received a VL‐test

Patient characteristics at date VL‐eligible	Eligible for VL‐test		Received VL‐test	
	N1	%^a^	N2	Coverage (%^b^)
Total (N)	21,400		18,182	85
ART regimen				
First‐line ART	21,004	98	17,832	85
Second‐line ART	396	2	350	88
POC site				
DHOS	6237	29	5112	82
Decentralized clinics				
NAMITAMBO	5761	27	5108	89
BILAL	4217	20	3716	88
MBULUMBUZI	2425	11	1985	82
NAMADZI	2760	13	2251	82
Months between ART initiation and date eligible for VL‐test				
≥6 to <12 months	7366	34	5761	78
≥12 to <24 months	2236	10	1945	87
≥24 months	11,798	55	10,476	89
Median years [IQR]	2.5	[0.5, 5.5]		
Sex				
Female	13,873	65	11,944	86
Male	7527	35	6238	83
Age				
<10 years	952	4	776	82
10 to 19 years	1020	5	864	85
20 to 39 years	10,626	49	8833	83
≥40 years	8802	41	7709	88
Median years [IQR]	38	[31, 46]		
Cumulative WHO stage				
I	5708	27	4718	83
II	5429	25	4738	87
III	6538	31	5642	86
IV	3509	16	2993	86
Missing	216	1	91	42
Prior CD4 cell count (cells/µL)				
0 to 199	2436	11	2060	85
200 to 349	4053	19	3539	87
350 to 499	4575	21	4090	89
≥500	7992	37	7093	89
No CD4	2344	11	1400	60
Median months^c^ [IQR]	9.3	[5.2, 16.6]		

^a^Column percentage; ^b^row percentage (N2/N1); ^c^median months between date sample taken for CD4 and eligibility.

### 3.1 VL coverage

During the study period, 85% (18,182/21,400) of eligible patients received a POC‐VL test. Namitambo and Bilal clinics had 89% VL coverage, whereas the DHOS coverage was 82%, similar to Mbulumbuzi and Namadzi clinics that implemented POC‐VL later (Table 1). For the 2 clinics with > 5000 eligible patients, the median time from eligibility to testing was 8.3 months (IQR: 2.8‐17.3), whereas for the three clinics with <5000 eligible patients, median time to testing was 5.5 months (IQR: 2.7‐11.0) (*p* < 0.01). VL coverage was similar among first and second‐line patients, but higher coverage was found for those on ART ≥24 months. Children and younger adults had slightly lower coverage than those ≥40 years old. Coverage was lowest among patients with no CD4, of which 44% (n = 1029) initiated ART under the treatment‐for‐all‐policy (after June 2016). This sub‐group was followed for a median of 2.8 months [IQR: 0.9‐5.6] between VL‐eligibility and last visit and had 40% VL‐coverage.

Among those VL‐tested, 89% (16,150/18,182) were identified as virally suppressed (VL < 1000 copies/mL) and 11% as suspected failures (VL ≥ 1000 copies/mL). Factors associated with suspected failure were longer duration on ART, male, younger age (children and young adults) and immune‐suppression (Table S1).

### 3.2 Turnaround‐time

**Figure 2:**
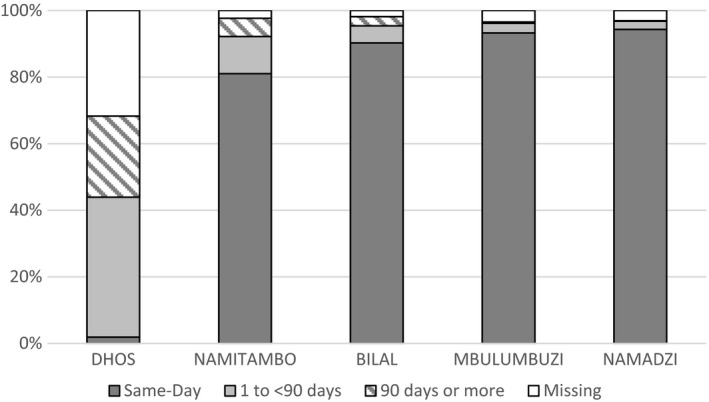
Turnaround time (in days) from sample collection to clinical review of VL test result by site.

Turnaround‐time could be analysed for 97% of tests requested in the decentralized clinics and for 68% of tests at DHOS (Figure 2). Missing data were due to incompletely filled lab request forms (missing review date and clinical decision making). In decentralized clinics, 88% of tests conducted had their results reviewed by clinicians on the same day as blood draw compared to 2% in the DHOS (*p* < 0.01), where the median TAT was 85 days [IQR: 83‐93].

### 3.3 Failure cascade

**Figure 3:**
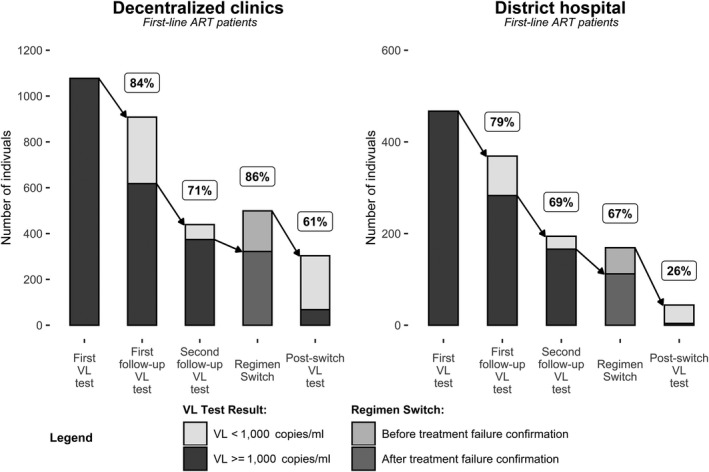
Treatment failure cascade among suspect‐failure patients on first‐line ART within 18 months of the first VL test, and the percent (%) who received a follow‐up VL test or a regimen switch according to the adapted MOH treatment failure algorithm.

Among first‐line suspected failures (n = 1544), 83% received a follow‐up test, of whom 29% (376/1277) had a VL < 1000 copies/mL (“suppressed”). Among those who had VL ≥ 1000 copies/mL on their follow‐up test (considered treatment failures per WHO definition) 70% (633/901) received a third test per the adapted MOH guidelines. Among those with follow‐up tests, 15% (93/633) were suppressed. Of those failing treatment (per adapted MOH guidelines), 80% (434/540) were switched to second‐line ART. A further 234 were switched prior to completing the failure algorithm; 54 were switched after the initial VL ≥ 1000, and 180 were switched after their first follow‐up test. Of all patients who switched, 52% (347/668) had a VL test post‐switch, among whom 79% (275/347) were suppressed. Switching rates and post‐switch VL‐testing coverage were significantly higher in the decentralized clinics compared to DHOS (*p* < 0.01) (Figure 3). Sensitivity analysis including 434 suspected failures with <18 months follow‐up showed similar failure cascade outcomes (Table S2). Retention in care at 18 months following suspect failure was 84% in decentralized clinics and 81% in DHOS (*p* = 0.16).

Among second‐line patients with an initial VL ≥ 1000 copies/mL, 76% (37/49) received a follow‐up test, of which 46% (17/37) suppressed. Among the non‐suppressed, 70% (14/20) received a third test and 64% (9/14) remained unsuppressed. None of the nine treatment failures were switched to a third‐line regimen during the study period due to limited access to resistance testing and third‐line ART. Both have since been introduced in the Chiradzulu cohort by MSF for second‐line failure patients.

Among all suspected failures, the median time between first and follow‐up VL‐tests or regimen switch was significantly shorter in the decentralized clinics (Table 2). As a result, the median time from initial VL ≥ 1000 copies/mL to regimen switch was 6.9 months in the decentralized clinics and 9.7 months in the DHOS. Once switched, the median time to a post‐switch test was 6.5 months and was similar across all sites.

**Table 2:** Median months between events in the failure cascade among first‐line and second‐line ART patients with suspected failure

Event 1	Event 2	Decentralized clinics		District Hospital	
		Median months [IQR]	N	Median months [IQR]	N
First VL‐test	First follow‐up VL‐test	3.3 [2.8, 4.4]	934	5.1 [3.8, 7.4]	380
	Second follow‐up VL‐test	6.6 [5.8, 8.1]	452	9.2 [7.6, 12.2]	195
	ART regimen switch	6.8 [5.6, 9.2]	499	9.7 [7.9, 13.2]	169
Treatment failure confirmation	ART regimen switch	0.9 [0.0, 2.8]	322	1.8 [1.0, 3.8]	112
ART regimen switch	Post‐switch VL‐test	6.5 [5.5, 8.2]	303	6.5 [4.9, 7.9]	44

ART, anti‐retroviral therapy; VL, viral load.

## 4 Discussion

This analysis presents the first programmatic outcomes of routine VL‐monitoring using a POC‐based approach in Malawi. In four years of implementation, encouraging VL‐testing coverage and efficient TATs were achieved, especially in decentralized clinics. Notably, despite no prior access to routine VL‐monitoring, 89% of patients tested had VL < 1000 copies/mL, nearly meeting the UNAIDS 90% viral suppression target for patients on ART 11. High VL suppression is likely related to continuous support provided by MSF at decentralized level, and is in line with good HIV care cascade coverage reported in 2012 for Chiradzulu District 40. Although follow‐up among suspected failure patients lead to overall good second‐line switching rates, we identified gaps in each step of the failure cascade.

Previous estimates paint a variable picture of the success of expanding VL‐testing in sub‐Saharan Africa, with 10%‐95% early uptake in national programmes (11%, Malawi 2014), and 40‐86% coverage achieved after 1 to 2 years of VL‐implementation in MSF supported facilities 17, 50, 51. More recent data from Malawi show increasing but still insufficient national scale‐up of VL‐testing (60% coverage) 52. Our testing‐coverage ranged between 82% and 89% in the 5 sites, likely higher in Namitambo and Bilal due to earlier VL‐access and direct support of MSF. Lower coverage rates were achieved in the DHOS, where MSF did not have a leadership role in VL testing, and in facilities (Mbulumbuzi and Namadzi) where VL was implemented later. Furthermore, time to first VL test took longer in the two early‐implementation sites, likely because of larger eligible cohorts (>5000). The commitment of managerial staff was also not always uniform across facilities and likely also impacted each site's individual performance. Similar coverage was achieved across patient characteristics (except somewhat lower rates for children and young adults, and patients <12 months on ART). Lower coverage observed among patients with no CD4 count was likely due to the very low VL‐coverage among patients who initiated ART more recently under the “treatment‐for‐all”‐policy with very short follow‐up time. Despite overall good coverage, achieving close to 100% should have been possible during the 3‐4 years‐period, highlighting remaining shortcomings, including clinicians' difficulties to effectively replace the long‐standing concept of CD4 monitoring with the new VL‐indicator.

Even when high VL‐testing coverage is achieved, good HIV treatment outcomes will remain out of reach if effective follow‐up of suspected failure patients does not occur. Our follow‐up testing rate among suspected failures (83%) was similar to recent findings in Lesotho (85%) and notably higher than rates reported previously in Swaziland (70%), Malawi (30%), Mozambique (35%) or Siaya County, Kenya (35%), 17, 50, 51, 53, 54, 55. Notably, less than one‐third of suspect failures had a suppressed VL at their first follow‐up test, likely corresponding to high levels of acquired first‐line drug resistance in this mature cohort where VL‐monitoring was only recently introduced, as reported in similar settings 17, 25, 34, 35, 50, 54, 56, 57. Access to routine VL‐testing, more frequent testing and a simple algorithm seem key to reducing treatment failure rates. In Swaziland where testing is annual, 62% of suspected failure patients were suppressed at their follow‐up test 51. In 2019, the MOH VL‐monitoring strategy in Malawi changed to annual testing instead of every 2 years 58. Modelling estimates that annual testing and regimen switching may prevent > 80% of transmitted resistance in Southeast Asia 59. Other benefits (like adherence support and the safeguarding of effective regimens) add to the appeal of early and more frequent VL‐testing 6.

Previous reports from sub‐Saharan African settings have consistently shown low and delayed switching from first‐ to second‐line ART (even when VL‐monitoring is present), which have been linked to poorer treatment outcomes, increased mortality and the risk of the development and transmission of resistance 8, 32, 60, 61, 62, 63. In our sites, 80% of first‐line treatment failures were switched to second‐line, an encouraging figure that is higher than in similar programmes (43%, Swaziland; 33%, Mozambique 50, 64). However, even 80% is insufficient. Clinicians may hesitate to switch patients who appear clinically well, while access to more expensive second or third‐line regimens remains often limited 8, 51, 60, 61. Staff often perceived that a detectable VL is synonymous with non‐adherence, and MOH guidelines until 2018 required good adherence before confirmatory VL test and switch 44, 45. Furthermore, the adapted algorithm which required two follow‐up tests with VL > 1000 copies/mL for failure confirmation likely delayed regimen switch unnecessarily. The majority (71%) had already been correctly identified as treatment failures at the first follow‐up test. Finally, 35% among those switched had not completed all steps of the failure cascade, indicating either difficulties in implementing the adapted three‐test failure algorithm or the non‐capture of VL‐tests in the electronic database. Encouragingly, in 2016 MOH guidelines lowered the failure confirmation threshold from 5000 to 1000 cps/mL 45, and since 2018 follow‐up VL‐testing for suspect failures is independent of good adherence 46, thus further streamlining VL scale‐up.

It remains critical to increase VL‐uptake and to ensure efficient use of VL results, including timely results and empowerment of clinicians and patients to make use of VL results 21. A key achievement of the POC‐VL approach in Chiradzulu was the reduction in the result turnaround‐time in the decentralized clinics, where MSF directly supported VL‐monitoring procedures by emphasizing to clinical staff and patients the importance of staying at the facility until the afternoon to allow same‐day results review. Using the most conservative TAT definition (blood collection to result communication) 88% of results were reviewed on the same day. In the DHOS, where the MOH administered VL monitoring, VL tests were performed on the same‐day as blood collection, but the clinicians did not stay long enough at the clinic for same‐day review, and results were provided at the patients' next scheduled visit (usually 3 months later, resulting in a median TAT of 85 days). Similarly, the failure cascade was significantly lengthened at the DHOS, with a median 9.7 months passing between initial high VL and switch to second‐line (vs. 6.9 months in the decentralized clinics). “Same‐day” results communication helps to link patients to EAC, supports failure follow‐up and reduces their time on failing ART. Moreover, instant results may increase clinicians' and patients' endorsement of VL‐monitoring. The same‐day results achieved in Chiradzulu's decentralized sites are thus a striking exception from TAT estimates reported from other sub‐Saharan and Malawian cohorts from 21 days to 3 months 17, 25, 51, 65. Notably, a recent clinical trial reported higher rates of retention, VL‐suppression and referral to decentralized care in patients who received POC‐VL‐testing 66. Yet, our findings (and others 17, 50, 53, 54, 56) indicate that providing access to the technology is not enough. Regular staff training, mentoring and VL “focal‐points” who monitor testing protocols are recommended. Staff rosters must be adjusted to optimize same‐day results. Monitoring systems with performance indicators should be implemented, ideally with integrated, automated systems that identify patients in need of VL‐testing 67. Creating demand for VL among clinicians and patients will be critical 20, 21, 35, 56, 68, 69, 70, 71. A recent modelling study predicted that switching patients on efavirenz‐based first‐line after only one high VL will significantly decrease mortality related to delayed switch 72, POC‐VL and same day results would further support this concept.

### 4.1 Strengths and limitations

Our analysis used real‐life programmatic data from a largely decentralized HIV programme, with a mature ART cohort that is typical of a high prevalence, sub‐Saharan African setting. We used a tailored data collection system that allowed reporting on every step of the VL‐testing cascade. Yet some limitations are present. Six percent of VL tests had to be excluded, possibly somewhat underestimating coverage and switching rates. Introduction to POC‐VL was staggered over a 15‐month period. Parallel introduction may have achieved higher coverage. The routine monitoring database did not capture EAC and we cannot describe the role of counselling on VL cascade outcomes. Longer term outcomes of suppressed patients, the impact of continuous VL‐monitoring on suppression rates, as well as further description of the feasibility of POC VL‐ testing are of interest but were outside the scope of our report. Successes and challenges experienced by MSF during routine VL implementation (at centralized laboratory or at point‐of‐care) have been reported elsewhere 35, 36. Finally, the continuous support and involvement of MSF over the past 15 years and during VL‐implementation, the relatively healthy cohort with high VL suppression at first‐ever VL test, as well as more complex VL‐testing algorithm that was used make our results less generalizable. A comparative study is required to directly assess the overall benefits of a POC‐based approach versus a centralized testing strategy.

## 5 Conclusions

Our findings indicate that routine point‐of‐care VL‐monitoring can achieve high testing coverage, same day results in decentralized clinics, and comparably good switching rates for first‐line failures. However, gaps at every step in the VL‐testing cascade remain a concern, including incomplete failure follow‐up and regimen switching. Close monitoring is recommended to identify potential gaps and directly guide programmes. VL technologies used at point‐of‐care can play a key role in bridging the gaps in access to VL‐monitoring and effective regimen switch in resource‐limited settings.

## Competing interests

The authors declare that they have no competing interests.

## Authors' contributions

SN, EP and BS designed the monitoring and analysis plan; MG and ES participated in the development of the monitoring and analysis plan; MG initiated and was the laboratory lead for POC‐VL implementation. AR supervised electronic data capture. MG, LW, JW and AR monitored and assured data quality; SN and EP provided technical support to monitoring and data quality; SN conducted the data management and undertook the analysis; SN and BS wrote the initial draft of the manuscript; All authors interpreted the findings and critically reviewed the manuscript and provided comments which were incorporated into subsequent drafts by SN and BS.

## Supporting information


**Table S1.** Association between patient characteristics at eligibility and suspected failure
**Table S2.** The failure cascade among first‐line ART patients with suspected failure
