# Supplementary material for: Point‐of‐care viral load monitoring: outcomes from a decentralized HIV programme in Malawi
Source: J Int AIDS Soc. 2019 Aug 23;22(8):e25387. doi: 10.1002/jia2.25387 (PMC6706700; doi:10.1002/jia2.25387)
Supplement: Supplementary file 1 — Table S1. Association between patient characteristics at eligibility and suspected failure Table S2. The failure cascade among first‐line ART patients with suspected failure [file JIA2-22-e25387-s001.docx]

Supplementary Table 1: Association between patient characteristics at eligibility and suspected failure

|  |  |  |  |  |  |  |  |  |  |  |  | |
| --- | --- | --- | --- | --- | --- | --- | --- | --- | --- | --- | --- | --- |
| Patient characteristics at date VL-Eligible | | Suspected Failure | | | | Bivariate Model | | Multivariate Model | | Sensitivity Analysis  Multivariate Model | | |
|  |  | N^1^ | n^2^ | %^3^ | p-value^4^ | cOR [95% CI] | p-value^5^ | aOR [95% CI] | p-value^5^ | aOR [95% CI] | p-value^5^ | |
| Total [N] | | 18182 | 2032 | 11 |  |  |  |  |  |  |  | |
| ART regimen | |  |  |  | <0.01 |  | <0.01 |  | 0.91 |  | 0.67 | |
|  | First-Line ART | 17813 | 1974 | 11 |  | 1.00 |  | 1.00 |  | 1.00 |  | |
|  | Second-Line ART | 369 | 58 | 16 |  | 1.49 [1.13, 1.99] |  | 1.02 [0.74, 1.39] |  | 1.07 [0.78, 1.47] |  | |
| POC site | |  |  |  | <0.01 |  | <0.01 |  | <0.01 |  | <0.01 | |
| Large Cohort | |  |  |  |  |  |  |  |  |  |  | |
|  | DHOS | 5112 | 660 | 13 |  | 1.00 |  | 1.00 |  | 1.00 |  | |
|  | NAMITAMBO | 5108 | 558 | 11 |  | 0.83 [0.74, 0.94] |  | 0.87 [0.76, 0.99] |  | 0.87 [0.76, 1.00] |  | |
| Small Cohort | |  |  |  |  |  |  |  |  |  |  | |
|  | BILAL | 3716 | 407 | 11 |  | 0.83 [0.73, 0.95] |  | 0.84 [0.73, 0.97] |  | 0.81 [0.70, 0.94] |  | |
|  | MBULUMBUZI | 1985 | 217 | 11 |  | 0.83 [0.71, 0.98] |  | 0.84 [0.71, 1.00] |  | 0.79 [0.65, 0.95] |  | |
|  | NAMADZI | 2251 | 190 | 8 |  | 0.62 [0.53, 0.74] |  | 0.67 [0.56, 0.81] |  | 0.67 [0.55, 0.81] |  | |
| Months between ART initiation and date eligible for VL test | |  |  |  | 0.39 |  | 0.39 |  | <0.01 |  | <0.01 | |
|  | ≥6 to <12 months | 5761 | 631 | 11 |  | 1.00 |  | 1.00 |  | 1.00 |  | |
|  | ≥12 to <24 months | 1945 | 204 | 11 |  | 0.95 [0.81, 1.13] |  | 1.28 [1.07, 1.54] |  | 1.28 [1.05, 1.55] |  | |
|  | ≥24 months | 10476 | 1197 | 11 |  | 1.05 [0.95, 1.16] |  | 2.05 [1.78, 2.35] |  | 2.03 [1.76, 2.35] |  | |
| Gender | |  |  |  | <0.01 |  | <0.01 |  | <0.01 |  | <0.01 | |
|  | Female | 11944 | 1163 | 10 |  | 1.00 |  | 1.00 |  | 1.00 |  | |
|  | Male | 6238 | 869 | 14 |  | 1.50 [1.37, 1.65] |  | 1.18 [1.06, 1.31] |  | 1.16 [1.05, 1.31] |  | |
| Age | |  |  |  | <0.01 |  | <0.01 |  | <0.01 |  | <0.01 | |
|  | <10 years | 776 | 314 | 40 |  | 9.61 [8.12, 11.37] |  | 14.85 [12.32, 17.88] |  | 14.19 [11.58, 17.40] | |  |
|  | 10-19 years | 864 | 274 | 32 |  | 6.59 [5.55, 7.78] |  | 8.70 [7.27, 10.41] |  | 8.99 [7.45, 10.85] | |  |
|  | 20-39 years | 8833 | 935 | 11 |  | 1.67 [1.50, 1.87] |  | 1.98 [1.76, 2.23] |  | 2.03 [1.80, 2.29] | |  |
|  | ≥40 years | 7709 | 509 | 7 |  | 1.00 |  | 1.00 |  | 1.00 |  | |

| Patient characteristics at date VL-Eligible | | Suspected Failure | | | | Bivariate Model | | Multivariate Model | | Sensitivity Analysis  Multivariate Model | | |
| --- | --- | --- | --- | --- | --- | --- | --- | --- | --- | --- | --- | --- |
|  |  | N^1^ | n^2^ | %^3^ | p-value^4^ | cOR [95% CI] | p-value^5^ | aOR [95% CI] | p-value^5^ | aOR [95% CI] | p-value^5^ | |
| Cumulative WHO stage | |  |  |  | <0.01 |  | <0.01 |  | <0.01 |  | | <0.01 |
|  | I | 4718 | 387 | 8 |  | 1.00 |  | 1.00 |  | 1.00 | |  |
|  | II | 4738 | 526 | 11 |  | 1.40 [1.22, 1.60] |  | 1.28 [1.10, 1.49] |  | 1.33 [1.14, 1.56] | |  |
|  | III | 5642 | 767 | 14 |  | 1.76 [1.55, 2.00] |  | 1.50 [1.29, 1.73] |  | 1.52 [1.29, 1.78] | |  |
|  | IV | 2993 | 341 | 11 |  | 1.44 [1.23, 1.68] |  | 1.31 [1.10, 1.56] |  | 1.32 [1.10, 1.60] | |  |
|  | Missing | 91 | 11 | 12 |  | 1.54 [0.81, 2.91] |  | 1.10 [0.55, 2.17] |  | 1.18 [0.36, 3.87] | |  |
| Prior CD4 cell count [cells/µL] | |  |  |  | <0.01 |  | <0.01 |  | <0.01 |  | | <0.01 |
|  | 0-199 | 2060 | 410 | 20 |  | 2.59 [2.26, 2.97] |  | 4.94 [4.19, 5.84] |  | 4.88 [4.13, 5.77] | |  |
|  | 200-349 | 3539 | 430 | 12 |  | 1.44 [1.27, 1.65] |  | 2.47 [2.14, 2.86] |  | 2.45 [2.12, 2.84] | |  |
|  | 350-499 | 4090 | 366 | 9 |  | 1.03 [0.90, 1.17] |  | 1.67 [1.45, 1.94] |  | 1.66 [1.43, 1.93] | |  |
|  | ≥500 | 7093 | 620 | 9 |  | 1.00 |  | 1.00 |  | 1.00 | |  |
|  | Missing | 1400 | 206 | 15 |  | 1.80 [1.52, 2.13] |  | 2.87 [2.32, 3.54] |  |  | |  |
| N^1^=Number with VL test; n^2^=number with VL>=1000 copies/ml; %^3^=percentage [n/N]; ^4^Pearon Chi-Square Test; ^5^ Likelihood Ratio Test | | | | | | | | | | | | |

**Footnote:**

Bivariate and multivariate logistic regression analyses was conducted to assess factors associated with suspected failure, and crude (cOR) and adjusted (aOR) odds-ratios are presented. The significance of factors was assessed by fitting all terms, and then successively dropping each from the adjusted model and conducting a likelihood ratio test. Further, a sensitivity analysis was conducted by excluding patients with missing CD4 cell count.

Amongst those VL-tested, 89% (16,150/18,182) 11% were identified as suspected failures (VL>1000 copies/mL). The proportion of suspect failures was higher among patients on second-line ART, though the effect was not significant in adjusted models. Suspect failure risk decreased with age, with double the odds in children and adolescents (<19 years) compared to those ≥40 years (aOR=14.9 and aOR=8.7, respectively). Likewise, a higher risk was associated with immunosuppression (aOR=4.9 when comparing CD4 <200 vs. ≥500 cells/µl). Higher risk was also associated with male gender, cumulative WHO stage >I, duration on ART >24 months, and missing CD4. Except for Namadzi [aOR=0.67], suspect failure risk was similar across sites. The results from the sensitivity analysis showed little difference from the multivariate model using the full dataset.

Supplementary Table 2. The failure cascade among first-line ART patients with suspected failure.

|  |  | **Patients on first-line ART with suspected failure retained in the analysis** | | | |  | **Sensitivity Analysis**  **All patients on first-line ART with suspected failure** | | | |
| --- | --- | --- | --- | --- | --- | --- | --- | --- | --- | --- |
|  |  | Decentralized Clinics | District Hospital | Total |  |  | Decentralized Clinics | District Hospital | Total |  |
|  |  | N=1077 | N=467 | N=1544 | p-value^1^ |  | N=1334 | N=640 | N=1974 | p-value^1^ |
| 1^st^ Follow up VL Test | |  |  |  |  |  |  |  |  |  |
|  | *% received VL test (n/N)* | 84%  (908/1077) | 79%  (369/467) | 83%  (1277/1544) | 0.01 |  | 80%  (1061/1334) | 73%  (465/640) | 77%  (1526/1974) | <0.01 |
|  | *% with a VL>1000 copies/mL (n/N)* | 68%  (618/908) | 77%  (283/369) | 71%  (901/1277) | <0.01 |  | 68%  (726/1061) | 76%  (355/465) | 71%  (1081/1526) | <0.01 |
| 2nd Follow up VL Test | |  |  |  |  |  |  |  |  |  |
|  | *% received VL test (n/N)* | 71%  (439/618) | 69%  (194/283) | 70%  (633/901) | 0.45 |  | 67%  (489/726) | 61%  (218/355) | 65%  (707/1081) | 0.05 |
|  | *% with a VL>1000 copies/mL (n/N)* | 85%  (374/439) | 86%  (166/194) | 85%  (540/633) | 0.90 |  | 85%  (417/489) | 87%  (189/218) | 86%  (606/707) | 0.62 |
| Switched to Second Line | |  |  |  |  |  |  |  |  |  |
|  | *% switched after suspected failure (n/N)* | 46%  (499/1077) | 36%  (169/467) | 43%  (668/1544) | <0.01 |  | 42%  (548/1334) | 32%  (204/640) | 38%  (752/1974) | <0.01 |
|  | *% switched after treatment failure (n/N)* | 86%  (322/374) | 67%  (112/166) | 80%  (434/540) | <0.01 |  | 84%  (349/417) | 65%  (122/189) | 78%  (471/606) | <0.01 |
| Post Switch VL test | |  |  |  |  |  |  |  |  |  |
|  | *% with post-switch VL test (n/N)* | 61%  (303/499) | 26%  (44/166) | 52%  (347/668) | <0.01 |  | 58%  (318/518) | 24%  (49/204) | 49%  (367/752) | <0.01 |
|  | *% with a VL<1000 copies/mL (n/N)* | 78%  (235/303) | 91%  (40/44) | 79%  (275/347) | 0.04 |  | 79%  (250/318) | 92%  (45/49) | 80%  (295/367) | 0.03 |
| ^1^ Pearson Chi-Square Test | | | | | | | | | | |
